# Correlating species and spectral diversities using hyperspectral remote sensing in early‐successional fields

**DOI:** 10.1002/ece3.2876

**Published:** 2017-04-06

**Authors:** Itiya P. Aneece, Howard Epstein, Manuel Lerdau

**Affiliations:** ^1^ Department of Environmental SciencesUniversity of VirginiaCharlottesvilleVAUSA

**Keywords:** band depth profiles, hyperspectral remote sensing, old‐field succession, plant pigments, plant species diversity, spectral first derivatives

## Abstract

Advances in remote sensing technology can help estimate biodiversity at large spatial extents. To assess whether we could use hyperspectral visible near‐infrared (VNIR) spectra to estimate species diversity, we examined the correlations between species diversity and spectral diversity in early‐successional abandoned agricultural fields in the Ridge and Valley ecoregion of north‐central Virginia at the Blandy Experimental Farm. We established plant community plots and collected vegetation surveys and ground‐level hyperspectral data from 350 to 1,025 nm wavelengths. We related spectral diversity (standard deviations across spectra) with species diversity (Shannon–Weiner index) and evaluated whether these correlations differed among spectral regions throughout the visible and near‐infrared wavelength regions, and across different spectral transformation techniques. We found positive correlations in the visible regions using band depth data, positive correlations in the near‐infrared region using first derivatives of spectra, and weak to no correlations in the red‐edge region using either of the two spectral transformation techniques. To investigate the role of pigment variability in these correlations, we estimated chlorophyll, carotenoid, and anthocyanin concentrations of five dominant species in the plots using spectral vegetation indices. Although interspecific variability in pigment levels exceeded intraspecific variability, chlorophyll was more varied within species than carotenoids and anthocyanins, contributing to the lack of correlation between species diversity and spectral diversity in the red‐edge region. Interspecific differences in pigment levels, however, made it possible to differentiate these species remotely, contributing to the species‐spectral diversity correlations. VNIR spectra can be used to estimate species diversity, but the relationships depend on the spectral region examined and the spectral transformation technique used.

## 1 Introduction

### 1.1 Remote sensing of diversity

Biodiversity can have numerous positive effects on the function of ecosystems. For example, it can affect ecosystem productivity by influencing resource‐use and promoting resource‐use efficiency (Cardinale et al., 2007; Gustafsson & Bostrom, 2011; Hooper & Vitousek, 1998; Symstad & Jonas, 2011; Wilsey & Potvin, 2000). It can also positively influence community stability by reducing fluctuations in production via compensatory effects (Gustafsson & Bostrom, 2011; Isbell, Polley, & Wilsey, 2009; Symstad & Jonas, 2011; Yachi & Loreau, 1999). In addition, biodiversity can affect infection resistance through increases in heterogeneity and thus dilution of hosts (Haas, Hooten, Rizzo, & Meentemeyer, 2011), and invasion resistance by again affecting resource‐use as well as by competitive effects (Cardinale et al., 2007; Gustafsson & Bostrom, 2011; Hooper & Vitousek, 1998; Scherber et al., 2010). Thus, conserving biodiversity is an important means for conserving ecosystem function.

Field methods are commonly used to estimate biodiversity in great detail at small spatial extents (Lengyel et al., 2008). However, these methods can be costly and time‐intensive, and difficult to scale up to larger spatial extents. Remote sensing can be used to collect information at vastly larger spatial extents more quickly and more cheaply per unit area than field sampling (Lengyel et al., 2008). It can also be combined with field data to more efficiently assess spatial and temporal distributions of biodiversity (Bradley & Mustard, 2006; Lengyel et al., 2008; Schmidt & Skidmore, 2001; Wilfong, Gorchov, & Henry, 2009; Zhang, Rivard, Sanchez‐Azofeifa, & Castro‐Esau, 2006) and to incorporate information at different spatial scales (Lengyel et al., 2008). Remote sensing has already been used to measure various indicators of species diversity, such as the normalized difference vegetation index (NDVI), biomass, land cover type, and heterogeneity in biomass and land cover (Foody & Cutler, 2003; Turner et al., 2003). The direct measurement of species diversity through species‐level characteristics is becoming possible with advances in satellite and aircraft technology, specifically the increases in spatial and spectral resolutions (Turner et al., 2003). Several researchers have been able to estimate species diversity and chemical diversity using remotely sensed data (Asner & Martin, 2008, 2011; Asner, Martin, Ford, Metcalfe, & Liddell, 2009; Asner, Martin, & Suhaili, 2012; Carlson, Asner, Hughes, Ostertag, & Martin, 2007; Feret & Asner, 2014; Rocchini et al., 2010).

Species diversity may be estimated by examining variability in spectral features (Asner & Martin, 2008; Rocchini et al., 2010), including those associated with pigments. Although pigment concentrations have traditionally been estimated using wet laboratory techniques, these procedures are labor‐ and time‐intensive, cannot be used for temporal analyses due to their destructive nature, need large numbers of samples for accurate representation of spatial variability (Blackburn, 2006), and can be inaccurate due to incomplete extractions, light‐absorbing impurities, and pigment instability (Merzlyak, Gitelson, Chivkunova, Solovchenko, & Pogosyan, 2003). In contrast, remote sensing, especially hyperspectral remote sensing, can be used to detect pigments quickly and nondestructively (Asner et al., 2007; Blackburn, 2006; Gamon & Berry, 2012; Gitelson, Keydan, & Merzlyak, 2006; Merzlyak et al., 2003; Yu, Lenz‐Wiedemann, Chen, & Bareth, 2014). We estimated the concentrations of carotenoids, anthocyanins, and chlorophylls, because they encompass the major groups of pigments in terrestrial plants (Delvin & Barker, 1971; Gitelson, Zur, Chivkunova, & Merzlyak, 2002), and the equations for estimating these three pigments are relatively well defined in the remote sensing literature (Gitelson et al., 2006; Merzlyak et al., 2003; Yu et al., 2014). Alongside determining useful features with which to estimate species diversity, we examined different spectral transformation techniques to assess their influence on diversity estimates.

### 1.2 Research objectives

Certain spectral features might be more useful for estimating biodiversity than others. The study of interspecific and intraspecific variability in these features will help elucidate the spectral regions most correlated with biodiversity. Assessing biodiversity can be important in early‐successional communities, where biodiversity and species composition may influence successional trajectory.

Here, we studied correlations between species diversity and spectral diversity in a temperate ridge and valley early‐successional ecosystem in north‐central Virginia. The Blandy Experimental Farm, our study site in Boyce, Virginia, includes chronosequences of successional fields inhabited by numerous exotic invasive species that control community biodiversity. These species can alter their surroundings, inhibiting the growth of other species and promoting their own growth both physically and chemically. In this study, we asked (1) whether species diversity was correlated with spectral diversity in secondary successional ecosystems in this region, (2) how these correlations differ by spectral region and spectral transformation technique, and (3) whether intraspecific and interspecific variabilities in pigments influence these correlations.

## 2 Methods

### 2.1 Study site

**Figure 1:**
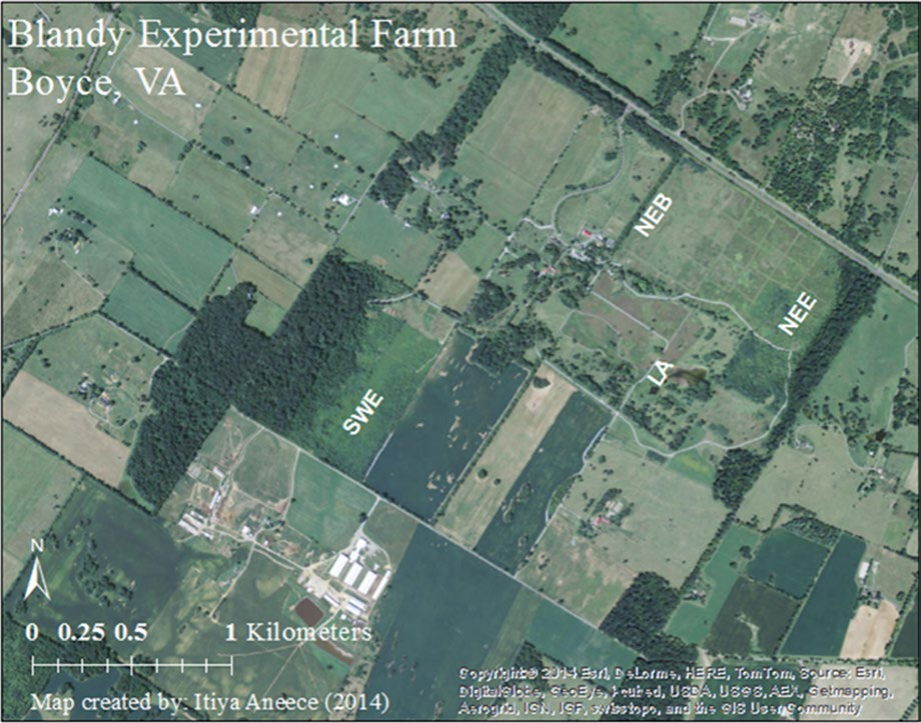
Blandy Experimental Farm in north‐central Virginia (39^*°*^09′N, 78^*°*^06′W) with study sites Southwest Early (SWE), Northeast Early (NEE), Northeast boundary (NEB), and Lake Arnold (LA)

We collected data at the Blandy Experimental Farm (BEF; Figure 1), which is located in the Shenandoah Valley in Clarke County Virginia at 39°09′N, 78°06′W (Wang, Shaner, & Macko, 2007). This 300‐ha biological field station has been owned by the University of Virginia (UVA) since 1926 and operated by the Department of Environmental Sciences at UVA since 1983 (Bowers, 1997). The field station includes 120 ha of pasture and cropland, 40 ha of woodland, the 60 ha Virginia State Arboretum, and 80 ha of old fields in early, middle, and late succession (Bowers, 1997). Each of two successional series (southwest and northeast) at the station is a set of former agricultural fields and contains an early‐, mid‐, and late‐successional field, abandoned in 2001 (Early 1), 2003 (Early 2), 1986 (Mid 1), 1987 (Mid 2), before 1910 (Late 1), and before 1920 (Late 2) (Wang, Epstein, & Wang, 2010). Spectral and species compositional data were collected from the two early‐successional fields and two additional field sites: Lake Arnold and a site at a field boundary near the northeast successional series referred to hereafter as the northeast boundary. The additional field sites were included because they were inhabited by an exotic invasive species not found in the other field sites. In this study, they are considered early successional due to the recency of disturbance. Vegetation at Lake Arnold consisted mostly of grasses and forbs, whereas the northeast boundary was composed of mostly grasses. Early‐successional stages in the field chronosequences mostly consisted of forbs and some grasses. For more information on species composition in these communities, see Aneece and Epstein (2015). Soils are deep colluvial and alluvial sediment from karst limestone, shale, and siltstone; study sites have well‐drained silt loam soil, of the soil Order Ultisol (Bowers, 1997). The average elevation of the BEF is 190 m, and slopes are <10% (Bowers, 1997). Mean annual temperature and precipitation are 11.8°C and 940 mm, respectively; the average growing season is 157 days with average annual primary productivity of approximately 1.0 kg/m^2^ in the successional fields (Bowers, 1997; Wang et al., 2010).

### 2.2 Field methods

**Figure 2:**
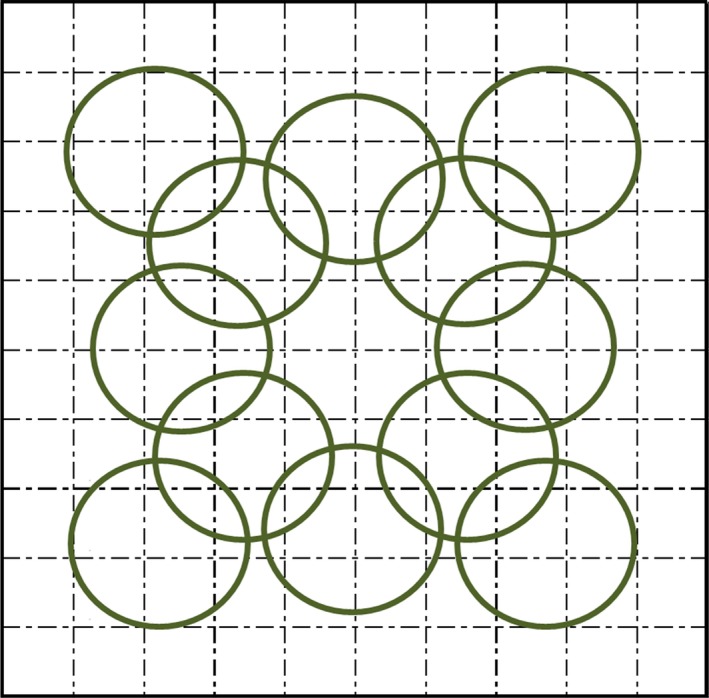
Layout of 5 × 5 m community plots. Circles represent spectral footprints taken from outside the plots and from the very center so as not to trample vegetation. Spectra from each corner of the plot, the center, and the middle of each edge for a total of 12 spectral footprints per plot were collected from approximately 2.5 m height from the ground so that the footprint was approximately 1.15 m in diameter. Vegetation surveys were conducted at each 0.5‐m interval within a plot for a total of 121 points (11 × 11) at the ground, understory, and canopy level

In the summer of 2014, we established three randomly placed 5 × 5 m community‐level plots at each early‐successional site, Lake Arnold, and the northeast boundary (Figure 1). Each community plot consisted of multiple species. From early June to late July, we collected community‐level spectral data from 350 to 1,025 nm using a PANalytical ASD Inc. FieldSpec^®^3, as this was the spectral range of the instrument, with a 25° field of view and a pistol grip. Spectra were normalized for light conditions with a Spectralon panel and viewing geometry was controlled for using a level on the pistol grip. The spectral range was defined by that able to be measured by the instrument. Spectra were collected from approximately 2.5 m height from the ground so that the footprint was approximately 1.15 m in diameter; footprint size was kept consistent by using this same height for all measurements. The relationship between footprint size and diversity was not determined in this research, but would be interesting to study; this size was used to obtain several subsamples within each plot that included spectral signature from multiple plant species. We collected spectra on cloud‐free days between 10 a.m. and 2 p.m. in each corner of the plot, in the center, and the middle of each edge for a total of 12 spectral footprints per plot (Figure 2). This system was used to maximize coverage without trampling vegetation and to correlate spectra with vegetation survey data. We conducted vegetation surveys on the 5 × 5 m grid at 0.5‐m intervals where grid lines intersected, recording species at the ground level, subcanopy, and canopy to assess the species diversity and species composition of the spectral footprints. As we knew which intersections from the vegetation surveys fell within each spectral footprint, we were able to match species compositions with spectral signatures.

In the summer of 2015, we collected leaf‐level spectra for pigment analysis from five of the dominant species in the community plots: *Achillea millefolium* (common yarrow), *Dactylis glomerata* (orchard grass), *Festuca rubra* (red fescue), *Solidago altissima* (tall goldenrod), and *Symphoricarpos orbiculatus* (coralberry) (Table 1, see Appendix S1 for species descriptions). All of these species have the potential to become invasive, especially in disturbed areas. Ten individual plants of each species were examined, except for *F. rubra*, of which five individual plants were sampled due to time and weather constraints. Three leaf samples were collected from each individual. We obtained leaf‐level spectra from detached leaves, which we wrapped in wet paper towels, put into zippered plastic bags, and stored on ice until measurements were taken within 20 min of detachment.

**Table 1:** Rank abundance of *Achillea millefolium* (common yarrow), *Dactylis glomerata* (orchard grass), *Festuca rubra* (red fescue), *Solidago altissima* (tall goldenrod), and *Symphoricarpos orbiculatus* (coralberry) in community plots at Blandy Experimental Farm in north‐central Virginia

	*A. millefolium*	*D. glomerata*	*F. rubra*	*S. altissima*	*S. orbiculatus*	Total # sps.
LACP4	–	–	9	–	–	9
LACP5	–	–	7	–	–	19
LACP6	–	–	8	–	–	23
NEBCP1	–	12	1	–	–	30
NEBCP2	–	12	2	–	–	22
NEBCP3	–	2	3	–	–	28
NEECP1	3	12	24	–	26	26
NEECP2	–	–	15	–	3	19
NEECP3	–	–	–	–	9	21
SWECP1	–	–	–	2	–	21
SWECP2	–	–	–	3	19	20
SWECP3	–	–	–	4	28	28

### 2.3 Statistical analysis

**Figure 3:**
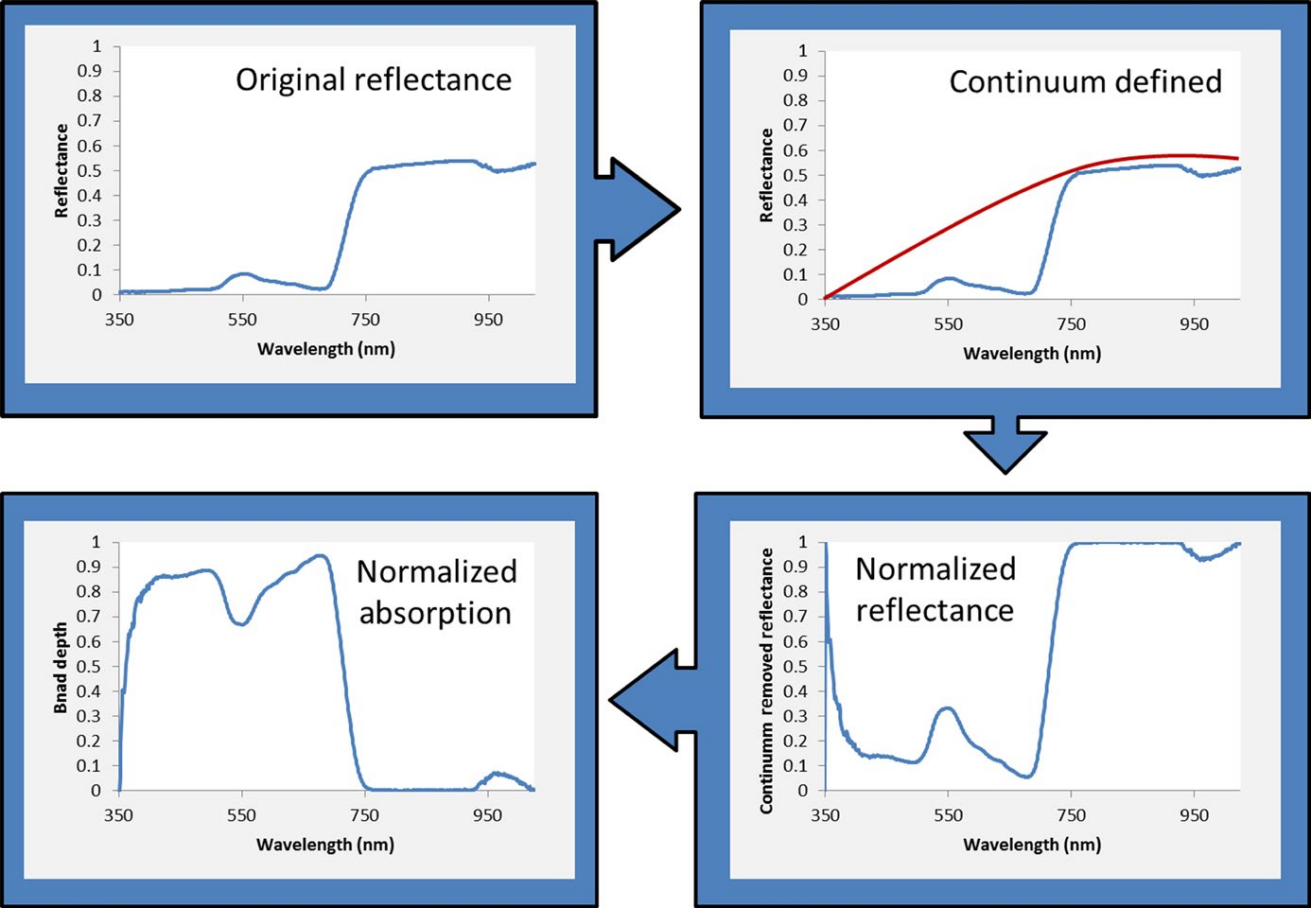
An illustration, using an average spectral profile from Dahurian buckthorn spectra, of calculating band depth (normalized absorption) from original reflectance using continuum removal. A continuum hull was established over the entire spectral profile. Then, the reflectance profile was subtracted from the continuum hull. The normalized reflectance was then subtracted from one to obtain normalized absorption

We used two spectral transformation techniques to examine whether the correlation between species and spectral diversities depends on the technique used. Such spectral transformation techniques are often used to enhance spectral features (Neumann, Forster, Kleinschmit, & Itzerott, 2016; Weber et al., 2008). We used band depth, or continuum removal, instead of original reflectance values to reduce noise from the sensor, atmosphere, soil background, topographic variation, and differences in albedo (Crowley, Brickey, & Rowan, 1989; Kokaly & Clark, 1999). Despite being normalized with a Spectralon, there was still variability in reflectance values at the near‐infrared shoulder by day and time of day. This was corrected for using band depth, which is used on dry and live plant matter to minimize variability due to differences in illumination and enhance spectral features (Thulin, Hill, Held, Jones, & Woodgate, 2012; Youngentob et al., 2011). To obtain band depth, a continuum hull was matched to the original spectral profile, and this continuum was removed to get normalized reflectance using ENVI (versions 5.0 and Classic, Exelis Visual Information Solutions, Boulder, Colorado). We then subtracted these continuum‐removed reflectance values from one to get the band depth profile (Figure 3). Continuum removal results differ by the spectral subset used; the spectra should be subset based on the features of interest (Harris Geospatial Solutions, 2017). In this study, we anchored the continuum hull to the red‐edge shoulder to minimize variability in the location of the red‐edge plateau due to differences in illumination and to enhance differences in the green peak. Band depth transformations are based on a priori information on the location of features of interest (like the green peak) and thus can be more stable (Shi, Zhuang, & Niu, 2004). With this a priori knowledge, continuum removal can be used to detect more subtle absorption features overlapping a continuum of absorptions (Huang, Turner, Dury, Wallis, & Foley, 2004).

We also assessed spectral diversity using first derivatives of the original reflectance profile as the second spectral transformation technique. First derivatives are often used in remote sensing to emphasize important spectral features, remove background noise, and lessen the influence of leaf water content (Inoue, Sakaiya, Zhu, & Takahashi, 2012; Ramoelo, Skidmore, Schlerf, Mathieu, & Heitkonig, 2011). They are assumed to decrease the influence of differences in illumination levels (Zhang et al., 2006), looking at changes in values relative to each other rather than absolute values. However, full‐band based transformations like first derivatives are highly influenced by the sampling environment and date of sampling and can emphasize wavelengths not traditionally associated with certain absorption features (Shi et al., 2004). When single regression analyses were conducted using original reflectance, the correlations between species diversity and spectral diversity were lower than when using band depth and first derivatives; thus, these spectral transformations were beneficial in correlating the two diversities.

**Figure 4:**
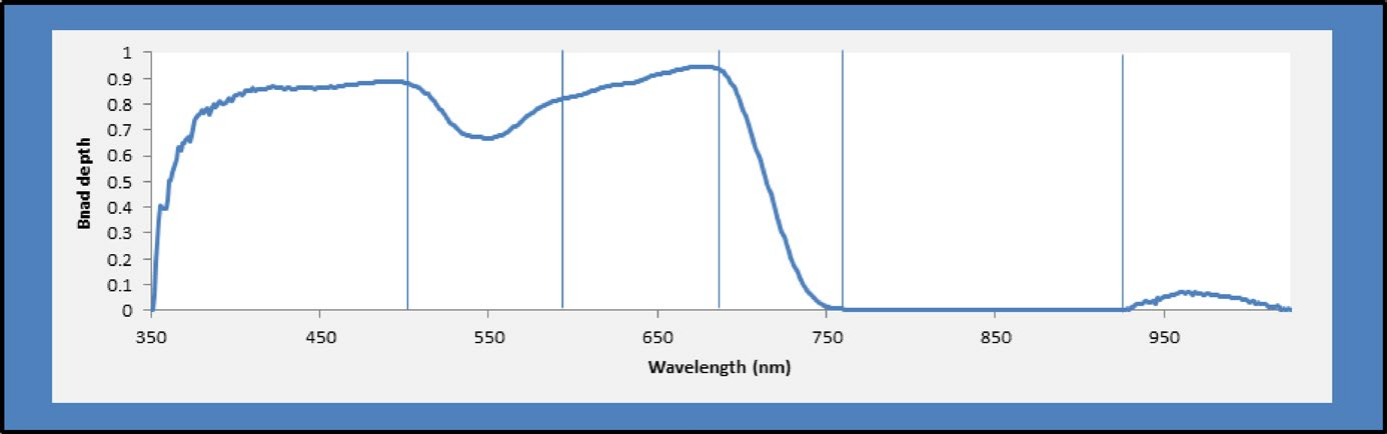
To quantify spectral diversity, band depth was divided into regions and areas under the curve calculated, and then standard deviations of the areas under the curve for respective plots were calculated

To quantify spectral diversity across an entire plot, we used standard deviations of areas under the band depth profile curve and the first derivative profile curve for the following regions corresponding with key spectral features: 350–499 nm (before the green peak), 500–589 nm (green peak), 590–674 nm (between green peak and red trough), 675–754 nm (red edge), 755–924 nm (near‐infrared plateau before water absorption feature), and 925–1,025 nm (water absorption feature; Figure 4). We calculated area under the curve as a way to incorporate information from all wavelengths in a spectral region without the problem of autocorrelation.

We calculated species diversity using the Shannon Diversity Index (Eq. (1)), where *p*_i_ is the proportion of species *i* and *n* is the number of species. To make a more direct comparison with spectral diversity, only the sampling points that were within the spectral footprints were included in calculating species diversity. We conducted single, multiple, and stepwise regression analyses in R (R Core Team, 2015) using the lm and stepAIC packages to assess the relationship between species and spectral diversities using spectra and vegetation surveys from the summer of 2014 for the early‐successional fields, Lake Arnold, and the northeast boundary.

(1) $$H^{\prime }=-\sum_{i=1}^{n}p_{i}*\text{ln}\left(p_{i}\right)$$

To assess whether the relationships between spectral diversity and species diversity may be influenced by the interspecific and intraspecific diversity of specific vegetation characteristics, we used the leaf‐level reflectance spectra of five species in the community plots to estimate pigment content of those five species and assess interspecific and intraspecific diversity of chlorophyll (Eq. (2)), carotenoid (Eq. (3)), and anthocyanin (Eq. (4)) levels using equations by Gitelson, Merzlyak, and Gritz (2003), Gitelson et al. (2006), and Gitelson, Merzlyak, and Chivkunova (2001), respectively, where *R*_770_, *R*_705_, *R*_515_, *R*_565_, *R*_550_, and *R*_700_ are reflectance values at 770, 705, 515, 565, 550, and 700 nm, respectively. These species were selected because they were present in many of the community plots and were prevalent in several of those plots [see figure 5 in Aneece and Epstein (2015)]. Reflectance spectra were used for these calculations because the equations are tailored toward reflectance measurements, rather than band depth. Chlorophyll, carotenoid, and anthocyanin levels were assessed using a nested analysis of variance (ANOVA) in SAS (Statistical analysis software, version 9.4, SAS Institute Inc., Cary, North Carolina) to compare intraspecific and interspecific pigment variability among *Achillea millefolium*,* Dactylis glomerata*,* Festuca rubra*,* Solidago altissima*, and *Symphoricarpos orbiculatus*, using among and within mean square and the *F* value. As parametric assumptions were not met, we used the nonparametric pairwise comparison Dwass, Steel, Critchlow–Fligner (DSCF) method to assess whether species were significantly different in terms of the following pigment estimates (SAS support, 2012):

(2) $$\text{Chlorophyll}=R_{770}\left(R_{705}^{-1}-R_{770}^{-1}\right)$$

(3) $$\text{Carotenoids}=R_{770}\left(R_{515}^{-1}-R_{565}^{-1}\right)$$

(4) $$\text{Anthocyanins}=R_{770}\left(R_{550}^{-1}-R_{700}^{-1}\right)$$

## 3 Results

Overall, spectral diversity was positively correlated with species diversity in several spectral regions across spectral transformations. We found slightly greater *R* ^2^ values with nonlinear relationships than with linear relationships in most cases, although the type of nonlinear relationship with the greatest *R* ^2^ value depended on the spectral region examined. Although nonlinear relationships had larger *R* ^2^ values than linear relationships, the differences in *R* ^2^ values were small; thus, interpretation of potential nonlinear relationships must be made with caution. For this reason, we used linear relationships to compare correlations between species diversity and spectral diversity across spectral transformations and spectral regions.

**Figure 5:**
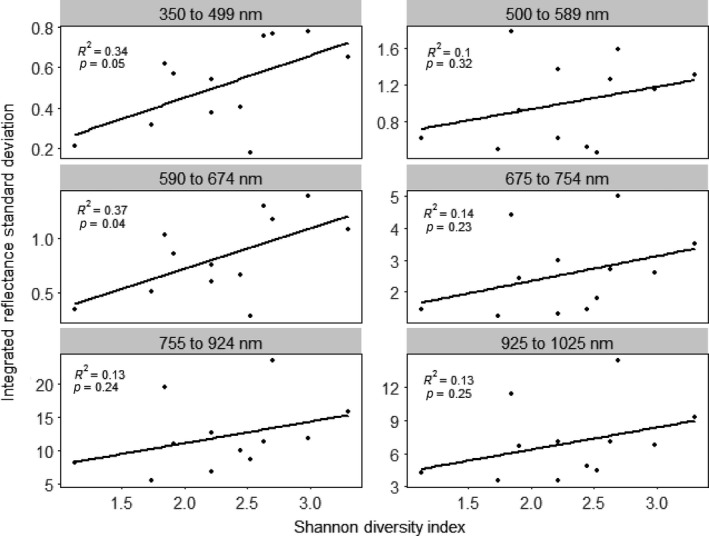
Correlations between species diversity and spectral diversity for six spectral regions using the area under the reflectance profile

**Figure 6:**
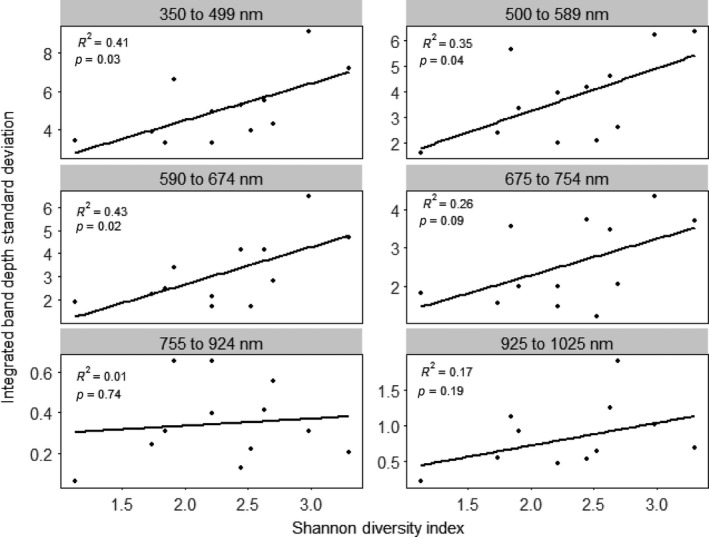
Correlations between species diversity and spectral diversity for six spectral regions using the area under the band depth profile

**Figure 7:**
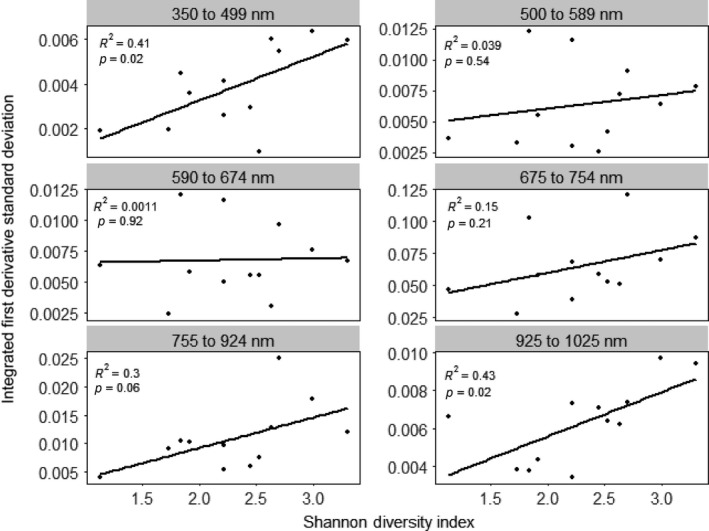
Correlations between species diversity and spectral diversity for six spectral regions using the area under the first derivative profile

Linear relationships using untransformed reflectance were not as strong as those using band depth and first derivatives (Figure 5). There were strong, significant, positive linear relationships between species diversity and spectral diversity using band depth in the summer of 2014 for the 350–499 nm wavelength region (*R* ^2^ = .41, *p* = .03), the 500–589 nm wavelength region (*R* ^2 ^= .35, *p* = .04), and the 590–674 nm wavelength region (*R* ^2^ = .43, *p* = .02), and a marginally significant positive relationship in the 675–754 nm wavelength region (*R* ^2^ = .26, *p* = .09; Figure 6). However, relationships between species diversity and spectral diversity were not significant in the 755–924 nm wavelength region (*R* ^2^ = .012, *p* = .74) or in the 925–1,025 nm wavelength region (*R* ^2^ = .17, *p* = .19). Using first derivatives instead of band depth, there was a strong positive correlation between spectral diversity and species diversity in the 350–499 nm wavelength region (*R* ^2^ = .41, *p* = .02; Figure 7) but no correlations in the 500–589 nm wavelength region (*R* ^2^ = .039, *p* = .54), the 590–674 nm wavelength region (*R* ^2^ = .0011, *p* = .92), and the 675–754 nm wavelength region (*R* ^2^ = .15, *p* = .21). There was a marginally significant positive correlation in the 755–924 nm wavelength region (*R* ^2^ = .30, *p* = .06) and a strong positive correlation in the 925–1,025 nm wavelength region (*R* ^2^ = .43, *p* = .02). Multiple regressions combining spectral diversity across regions to estimate species diversity revealed lower *R* ^2^ values than when considering individual regions for both spectral transformation techniques and thus were not further considered. Given the small sample size in conducting these correlation analyses, we repeated them on 20 random samples each consisting of 8 of 12 plots. The average *R* ^2^ values across these 20 samples led to the same patterns in comparing *R* ^2^ values across spectral regions and spectral transformation techniques as when using all 12 plots. Thus, the comparisons are reliable despite sample size.

Although the first derivative and band depth transformations resulted in larger *R* ^2^ values across spectral regions in the single regressions, untransformed reflectance had larger *R* ^2^ values than the spectral transformations using multiple linear regression analyses (Table 2). This was also supported by the stepwise multiple regression analyses (Table 3). Looking across stepwise regression models, the most influential spectral regions were 500–589 nm, 590–674 nm, and 925–1025 nm, which supports the results of the single regressions when considering only relationships significant at the level of *p* = .05.

**Table 2:** A comparison between multiple regression results of linear and nonlinear relationships between species and spectral diversities across different spectral transformations and spectral regions

Transformation	Relationship type		
	Linear	Logarithmic	Exponential
Reflectance	0.56 (0.10)	0.56 (0.10)	0.43 (0.18)
First derivative	0.23 (0.27)	0.29 (0.28)	0.30 (0.27)
Band depth	0.14 (0.39)	0.19 (0.36)	0.28 (0.29)

*R* ^2^‐value (*p*‐value). Multiple regressions with 2nd‐order polynomials were not possible due to sample size.

**Table 3:** A comparison of stepwise regression results across relationship types and spectral transformations

Transformation	Relationship	Spectral Region (nm)						*R* ^2^‐value (*p*‐value)
		350–499	500–589	590–674	675–754	755–924	925–1,025	
Reflectance	Linear		+	+	+	+	+	.63 (.04)
	Logarithmic		+	+	+	+	+	.61 (.05)
	Exponential		+	+	+	+	+	.52 (.09)
First derivative	Linear		+	+			+	.55 (.03)
	Logarithmic					+	+	.59 (.01)
	Exponential		+	+			+	.55 (.03)
Band depth	Linear		+	+	+			.37 (.09)
	Logarithmic	+					+	.44 (.03)
	Exponential	+	+	+	+	+		.38 (.16)

Stepwise regressions with 2nd‐order polynomials were not possible due to sample size. The (+) signs indicate spectral regions that were retained in the regressions.

**Figure 8:**
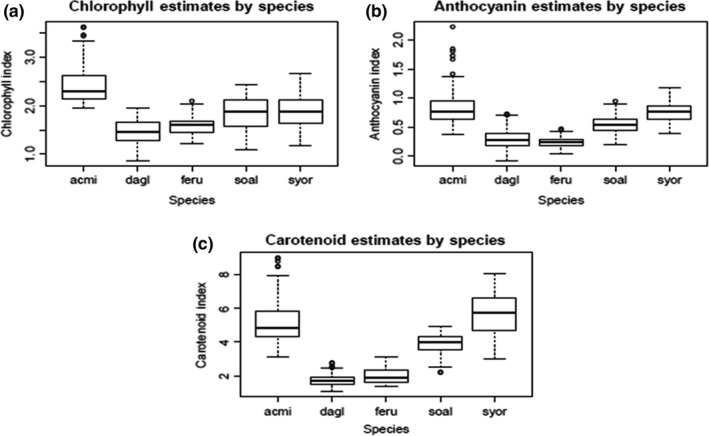
Estimates of (a) chlorophylls, (b) anthocyanins, and (c) carotenoids for *Achillea millefolium* (acmi), *Dactylis glomerata* (dagl), *Festuca rubra* (feru), *Solidago altissima* (soal), and *Symphoricarpos orbiculatus* (syor) using ground‐level hyperspectral data

The analysis of variance for pigment estimates revealed that there was greater interspecific variability than intraspecific variability in terms of all three pigment types; however, within‐species variability was proportionally greater in chlorophyll than in carotenoid and anthocyanin estimates (Table 4). This is concluded based on the F value, which is the ratio of variance among species (among mean square) to variance within species (within mean square). This greater intraspecific variability may account for some of the lack of correlation between spectral diversity and species diversity in the red trough region. Although there is greater intraspecific variability in chlorophyll than the other pigments, interspecific variability is still greater than intraspecific variability, leading to significant differences by species for all three pigments (Figure 8). In terms of anthocyanins and carotenoids, all species were significantly different (*p* *<* .001) except for *A. millefolium* vs. *S. orbiculatus* and *D. glomerata* vs. *F. rubra*. In terms of chlorophyll, all species were significantly different except for *D. glomerata* vs. *F. rubra* and *S. altissima* vs. *S. orbiculatus*.

**Table 4:** ANOVA results comparing among and within variance in pigment estimates by species

Pigment	Among mean square	Within mean square	*F* value
Chlorophylls	12.454	0.1059	117.59
Anthocyanins	5.8774	0.0467	125.87
Carotenoids	260.21	0.8093	321.52

## 4 Discussion

As biodiversity can influence ecosystem function and stability, its study is clearly important to aid conservation efforts. Small‐scale analyses of diversity are possible using field methods, but remote sensing is typically needed for assessment at large spatial scales. To estimate species diversity, we may be able to use diversity of spectral features such as those associated with pigments. In this article, we asked whether species diversity and spectral diversity were correlated, especially diversity in pigment features; we also asked whether spectral transformation techniques influenced the correlation between species and spectral diversities. Nonlinear relationships had only slightly larger *R* ^2^ values than linear relationships; thus, their biological meaning must be interpreted with care. Thus, we focused on the linear relationships to compare spectral transformations and spectral regions in terms of the relationship between spectral diversity and species diversity. When using band depth, we found that the two were strongly linearly positively correlated in the visible region (350–674 nm), weakly linearly positively correlated in the red‐edge region (675–754 nm), and uncorrelated in the near‐infrared region (755–1,025 nm). When using first derivatives, we found a strong linear positive correlation in the 350–499 nm region, but no correlation in the other visible ranges (500–674 nm) or in the red‐edge region (675–754 nm); however, we found positive linear correlations in the near‐infrared region (755–1,025 nm). Therefore, the method of spectral transformation and the spectral regions considered will influence the ability to estimate species diversity using spectral diversity.

### 4.1 Visible region

The 350–499 nm region had a strong positive correlation with H' using band depth and first derivatives, suggesting that this region has large interspecific variability. Using band depth, there were also strong positive correlations in the rest of the visible region. However, there were no other correlations in the visible region when using first derivatives. This may be because first derivatives have been found to exaggerate noise due to environmental variation in this region, such as aerosol content and differences in illumination, and increase intraspecific spectral reflectance variability (Zhang et al., 2006).

### 4.2 Near‐infrared region

The lack of correlation in the near‐infrared region when using band depth may be due to the fact that the continuum removal applied for band depth calculations drastically reduced variability in the near‐infrared plateau; this reduction may mask variability in the near‐infrared plateau that may be caused by interspecific differences. Therefore, it may be better to use derivatives to correlate species diversity and spectral diversity in this region. Indeed, when using first derivatives, there was a strong positive correlation between spectral diversity and species diversity in the 755–924 nm wavelength region (*R* ^2^ = .30, *p* = .06) and the 925–1,025 nm wavelength region (*R* ^2^ = .43, *p* = .02; Figure 7).

### 4.3 Red trough and red‐edge regions

The most interesting result perhaps is a weak correlation in the red‐edge region using band depth and the lack of correlation using first derivatives, due to greater intraspecific variability versus interspecific variability in this region. Variability in the red‐edge region may be due to differences in the red trough or differences in the near‐infrared plateau; however, since differences in the near‐infrared plateau are minimized while using band depth, the differences are likely in the red trough. To determine how there might be greater interspecific variability in most of the visible region yet greater intraspecific variability in the red‐edge region, especially the red trough, the absorption peaks of different pigments were considered. Chlorophyll a and b peaks are in the visible and red trough regions, and anthocyanin and carotenoid peaks occur in the visible region [for more detailed absorption peak locations, see Jensen (2007)]. The intraspecific variability in the red‐edge region (675–754 nm) may be due to intraspecific variability in chlorophyll content, which may be more plastic and more sensitive to environmental factors than other pigments. In contrast, carotenoid and anthocyanin content may have greater interspecific variability than intraspecific variability. This may be because anthocyanin content and carotenoid content are highly influenced by genetics (Ficco et al., 2014; Fournier‐Level et al., 2009), whereas chlorophyll content is influenced by both genetics and environmental conditions and stressors (Cao, 2000; Malyshev et al., 2016).

### 4.4 Nonlinear relationships

As mentioned above, nonlinear relationships had slightly larger *R* ^2^ values and thus the relationship between spectral diversity and species diversity may not be linear in all spectral regions and transformations. In several cases, the exponential relationship was stronger than the linear relationship, with an *R* ^2^ value maximum difference of approximately .02 (Table 5). This may mean that as species diversity increases, spectral diversity increases to an even greater extent, perhaps due to intraspecific variability. In one case, the logarithmic relationship was stronger, again with an *R* ^2^ difference of .02. This may indicate saturation of spectral diversity with an increase in species diversity. In one case, a 2nd‐order polynomial relationship was stronger by an *R* ^2^ difference of .06; however, this seems highly influenced by one point. Considering the small *R* ^2^ differences between these nonlinear and linear relationships, the meaning of the nonlinear relationships must be interpreted with caution.

**Table 5:** A comparison between different linear and nonlinear relationships between species diversity and spectral diversity across different spectral transformation techniques and spectral regions

Transformation	Region	Relationship type			
		Linear	2nd‐order polynomial	Logarithmic	Exponential
Reflectance	350–499 nm	0.34 (0.05)			0.36 (0.04)
	500–589 nm				
	590–674 nm	0.37 (0.04)			0.38 (0.03)
	675–754 nm				
	755–924 nm				
	925–1,025 nm				
First Derivative	350–499 nm	0.41 (0.02)	0.48 (0.02)		0.41 (0.02)
	500–589 nm				
	590–674 nm				
	675–754 nm				
	755–924 nm				
	925–1,025 nm	0.43 (0.02)	0.40 (0.04)	0.37 (0.04)	0.43 (0.02)
Band Depth	350–499 nm	0.41 (0.03)		0.43 (0.02)	
	500–589 nm	0.35 (0.04)		0.35 (0.04)	0.37 (0.04)
	590–674 nm	0.43 (0.02)		0.40 (0.03)	
	675–754 nm				
	755–924 nm				
	925–1,025 nm				

*R* ^2^‐value (*p*‐value). Only relationships significant to *p* = .05 are included.

#### 4.4.1 Multiple and stepwise regressions

Although first derivative and band depth transformations had stronger correlations between spectral diversity and species diversity than untransformed reflectance in the single regression analyses, multiple and stepwise regressions combining all spectral regions revealed stronger correlations using reflectance. This might be because all spectral regions had slight positive correlations between spectral diversity and species diversity using reflectance while only some regions had strong positive correlations when using first derivatives and band depth. This is demonstrated in the stepwise regression analyses, in which almost all regions were retained as important when using reflectance while only some regions were retained as important when using first derivatives and band depth. When looking across all stepwise regressions, the spectral regions that were most often deemed important were 500–589 nm (green peak), 590–674 nm (red trough), and 925–1,025 nm (near‐infrared plateau). Thus, future studies may be able to focus on these regions when estimating species diversity using spectral diversity.

### 4.5 Species pigment comparisons

To assess intraspecific and interspecific differences in pigment contents, we used spectra of five dominant species in the community plots to calculate indices estimating the amounts of chlorophyll (Eq. (2)), carotenoids (Eq. (3)), and anthocyanins (Eq. (4)) in the leaves. There was greater intraspecific variability in chlorophyll than in carotenoids and anthocyanins (Table 4); however, there was overall greater interspecific variability than intraspecific variability. Species were significantly different in terms of all spectral pigment estimates (Figure 8). *A. millefolium* had greater chlorophyll content than did *S. orbiculatus* and *S. altissima*, which had greater chlorophyll content than did *D. glomerata* and *F. rubra*. *A. millefolium* and *S. orbiculatus* had greater anthocyanin content than *S. altissima*, which had greater anthocyanin content than *D. glomerata* and *F. rubra*. In contrast, Veres et al. (2006) found that *Festuca pseudovina* had higher xanthophyll content than *A. millefolium*. In this study, *A. millefolium* and *S. orbiculatus* had greater carotenoid content than *S. altissima*, which had greater carotenoid content than *D. glomerata* and *F. rubra*. Similarly, Veres et al. (2006) found that out of the monocots they tested, *Festuca pseudovina* had the lowest carotenoid content, and of the dicots tested, *A. millefolium* had the greatest carotenoid content. Carotenoid content and composition of different carotenoids can vary by environment and have high interspecific variation (Veres et al., 2006).

There may be several reasons why *Festuca rubra* and *Dactylis glomerata* had low levels of photoprotective pigments. Grass leaves have high Si content, which might help them reflect UV‐B radiation and thus not need as much photoprotection from pigments (Deckmyn & Impens, 1999). Out of *Festuca arundinacea*,* Festuca rubra*,* Lolium perenne*, and *Poa pratensis*, Zhang and Ervin (2009) found that *F. rubra* had the greatest tolerance to UV‐B. This higher tolerance may be due to narrower leaves and thick waxy cuticles (Zhang & Ervin, 2009). Narrow leaves can lead to a reduction in boundary layer growth, thus reducing leaf temperature in high light conditions (Letts, Flannagan, Van Gaalen, & Johnson, 2009). When treating *F. rubra* and *D. glomerata* with increasing levels of UV‐B, Deckmyn and Impens (1999) found that there was an increase in protective pigments in *D. glomerata*, but not in *F. rubra*. This implies that *F. rubra* may have a different way of dissipating excess energy such as antioxidant activity and activation of hormones that cue defense mechanisms (Zhang & Ervin, 2009).

Another reason these species were significantly different from each other in terms of pigment levels may be that they are from different plant functional types (two grasses, two forbs, and one shrub). Forbs have lower foliar support costs than shrubs, which need to invest more in woody biomass growth; therefore, forbs may have greater leaf dry mass per unit area than do woody species (Niinemets, 2010). This greater ability to invest in leaves may explain the high chlorophyll levels of *A. millefolium* compared with those of *S. orbiculatus*, although those of *S. altissima* were just as low.

These plants also differ in shade tolerance; *S. altissima* is less shade tolerant than *S. orbiculatus* and *A. millefolium*, which are less shade tolerant than *D. glomerata* and *F. rubra*. Shade‐tolerant species usually have lower leaf dry mass per unit area and greater specific leaf area to intercept more light in the shade (Niinemets, 2010). These leaves with high specific leaf area have greater longevity but lower net photosynthesis levels and lower photosynthetic nitrogen‐use efficiency, because of greater allocation to nonphotosynthesizing cell wall material and large vein networks over photosynthetic machinery (Johnson & Tieszen, 1976; Niinemets, 2010). In this study, the two most shade‐tolerant species also had the lowest concentrations of pigments.

For these pigment analyses, leaf‐level spectra were used to examine only photosynthetic tissue and thus get a more accurate representation of photosynthetic machinery. However, diversity correlations were made using spectra that included both photosynthetic and structural elements. Structural signatures are more prevalent in the shortwave‐infrared region than the visible and near‐infrared regions (Mahlein, 2011), but a component of structure is leaf angle distribution, which in turn affects signatures in the visible and near‐infrared regions. Thus, some of the variability in the correlation analyses may be due to the structural component of species diversity. The variability in pigment indices across species, combined with structural variability, shows the utility of hyperspectral data for assessing species diversity across landscapes.

Overall, band depths of visible range values within the 350–674 nm region can be used to estimate species diversity. This finding of a correlation between spectral diversity and species diversity supports prior research (Asner & Martin, 2011; Asner et al., 2007, 2009, 2012; Carlson et al., 2007; Feret & Asner, 2014; Rocchini et al., 2010). However, other methods of spectral transformation might need to be implemented to use the near‐infrared region for estimating species diversity. Although there are several methods at the satellite level to classify vegetation and estimate diversity, these methods mostly use reflectance values. This research examined spectral transformation techniques at the ground level to illustrate the benefits of using band depth and first derivatives over original reflectance to estimate species diversity. Additionally, variability in the red‐edge region may be due to intraspecific variability in chlorophyll *a* and *b* content rather than differences in species composition. Species plasticity in pigment levels also needs to be considered when analyzing species discriminability; however, this difference in pigment levels across species supports the possibility of discriminating species spectrally. Species discrimination and diversity estimation at the satellite level will be challenging because of more complex landscapes. One such challenge is the presence of nonvegetated surfaces, which need to be masked out, perhaps using a normalized difference vegetation index value threshold. Another challenge is presented when there is a high degree of structural diversity within a single species, such as with clonal plants. This structural diversity and its effects on spectral diversity would be useful to understand. Additionally, there are several scales of diversity; a study of how spectral diversity captures alpha and beta diversity would also be useful. This may be possible with airborne and satellite‐based imagery that has high spatial and at least moderate spectral resolution. Soil signatures in areas with low vegetation may also pose challenges; variability in spectral signatures in such areas may be due to differences in soil types, textures, and/or moisture levels as well as differences in vegetation.

Despite these challenges, the ability to estimate species diversity using spectral diversity would facilitate several practical tasks. For example, the assessment of spectral diversity in a particular region over time could provide a rapid and reliable way to estimate changes in species diversity over time. Thus, remote sensing can be used to estimate diversity and aid conservation efforts at large spatial extents; however, methods used to estimate diversity must be chosen and interpreted carefully.

## 5 Conclusions

The correlation between species diversity and spectral diversity depends on the spectral region examined and the spectral transformation technique used. Using band depth, regression analyses revealed positive correlations between spectral diversity and species diversity in the visible ranges of 350–499 nm (*R* ^2^ = .41, *p* = .03), 500–589 nm (*R* ^2^ = .35, *p* = .04), and 590–674 nm (*R* ^2^ = .43, *p* = .02), slight positive correlation in the red‐edge range of 675–754 nm (*R* ^2^ = .26, *p* = .09), and no correlation in the near‐infrared ranges of 755–924 nm (*R* ^2^ = .012, *p* = .74) and 925–1,025 nm (*R* ^2^ = .17, *p* = .19). Using first derivatives, we found a strong positive correlation in the visible range of 350–499 nm (*R* ^2^ = .41, *p* = .02), but no correlations in the visible ranges of 500–589 nm (*R* ^2^ = .039, *p* = .54) and 590–674 nm (*R* ^2^ = .0011, *p* = .92); we found no correlation in the red‐edge region (*R* ^2^ = .15, *p* = .21) and positive correlations in the near‐infrared ranges of 755–924 nm (*R* ^2^ = .30, *p* = .06) and 925–1,025 nm (*R* ^2^ = .43, *p* = .02). The lack of correlation in the visible region using first derivatives may be because first derivatives exaggerate spectral noise in the visible region. The lack of correlation in the near‐infrared region using band depth may be because band depth minimizes variability in the near‐infrared region, thus dampening interspecific differences. The lack of correlation in the red edge may be partially due to the greater intraspecific variability of chlorophyll content over content of other pigments. This variability can be expressed in the red trough region, at the base of the red edge, dampening interspecific differences and thus lessening the correlation between species diversity and spectral diversity.

## Author Contributions

I.A. and H.E. designed the study. I.A. collected and analyzed data. I.A., H.E., and M.L. worked on the manuscript.

## Conflicts of Interest

The authors declare no conflict of interest. The funding sponsors had no role in the design of the study; in the collection, analysis, or interpretation of the data; in the writing of the manuscript; and in the decision to publish the results.
