# Supplementary material for: Correlating species and spectral diversities using hyperspectral remote sensing in early‐successional fields
Source: Ecol Evol. 2017 Apr 6;7(10):3475–88. doi: 10.1002/ece3.2876 (PMC5433985; doi:10.1002/ece3.2876)
Supplement: Supplementary file 1 [file ECE3-7-3475-s001.docx]

**(A) Appendix S1 {Species Descriptions}**

*Achillea millefolium* is a perennial herb with one to ten stems 20-100 cm tall, coming from a fibrous rhizome (Hurteau, 2001; Cummins, 2014). Its leaves are lanceolate, bipinnately dissected, 0.5-3 cm wide, 3-15 cm long, and can be pubescent (Hurteau, 2001). The inflorescence is a corymb with 10-20 whitish to yellowish-white ray flowers (Hurteau, 2001). It can grow in disturbed, well-drained soils in grasslands and open forests, and is drought tolerent; it persists from May to June (Hurteau, 2001). *A. millefolium* is not tolerant of dense shade, and has a palatability of fair to poor (Aleksoff, 1999).

*Dactylis glomerata* is a cool season perennial C3 bunchgrass, and is one of the earliest grasses to emerge in spring; it can grow 50-120 cm tall (Bush et al., 2012; Barbehenn and Bernays, 1992). Its leaves are 2-8 mm wide and 20-30 cm long, and its panicles are 5-20 cm long with laterally compressed spikelets of 2-5 flowers in dense one-sided clusters (Bush et al., 2012). *D. glomerata* does not reproduce vegetatively, but still has the potential to become invasive (Bush et al., 2012). It grows well in moderate to well-drained slightly basic to acidic soils (pH 5.8-7.5), that can vary in texture from clay to gravelly loam, and in depth from shallow to deep; however, it cannot tolerate salinity or high soil moisture (Bush et al., 2012). *D. glomerata* can tolerate cold winters when snow is present for insulation, high summer temperatures and humidity, as well as shade. It has a high palatability (Sullivan, 1992).

*Festuca rubra* is a C3 perennial cool season grass that also starts growing early in the spring with a high growth rate in late summer (Barbehenn and Bernays, 1992; St. John et al., 2012). Its leaves are mostly basal, 5-15 cm long and 1-2 mm wide; the inflorescence is a narrow panicle 3-20 cm long with 3-10-flowered spikelets (St. John et al., 2012). *F. rubra* grows rhizomatously, and can become invasive especially in disturbed areas (St. John et al., 2012). It is shade and salt tolerant, and grows in soils of varying texture from sandy to gravelly, from moist meadows to disturbed areas; it can grow in soils that have low fertility and varying pH, from acidic to slightly alkaline (pH 4.5-7.5) (St. John et al., 2012). *F. rubra* has fair palatability (St. John et al., 2012).

*Solidago altissima* is a perennial forb, 1-2 m tall (Citizens United, 2010). Its leaves are 15 cm long, 2 cm wide, linear-lanceolate in shape, pubescent on the underside, and scabrous on the upper surface of the leaf; its inflorescence is a terminal pyramidal panicle, 15 cm broad and 20 cm tall with yellow flowers, blooming from August to November (Tenaglia, 2007). *S. altissima* inhabits dry open spaces, fallow fields, prairies, rocky outcrops, open woods, thickets, wastelands, roadsides, and railroad corridors (Citizens United, 2010; Tenaglia, 2007). Within the fields at the BEF, this species forms extensive monocultural stands.

*Symphoricarpos orbiculatus* is a branching shrub, 0.66 to 1.33 m tall (Hilty, 2015). Its leaves are 5 cm long, 3 cm wide, oval-ovate in shape with an upper surface that is glabrous to slightly pubescent (Hilty, 2015). Flowers are greenish yellow and approximately 0.64 cm long; berries are reddish purple and approximately 0.64 cm long (Hilty, 2015). *S. orbiculatus* grows in partial sun, moist to dry soil, loamy to rocky in texture; it inhabits thin rocky woodlands, woodland openings and borders, disturbed areas, thickets, and limestone glades (Hilty, 2015). It is very palatable to deer (Hilty, 2015).

References:

Barbehenn, R. and Bernays, E. (1992) Relative nutritional quality of C3 and C4 grasses for a graminivorous lepidopteran, Paratrytone melane(Hesperiidae). *Oecologia*, **92**, 97–103.

Bush, T., Ogle, D., St. John, L., Stannard, M., and Jensen, K. (2012) *Plant guide for orchardgrass (Dactylis glomerata)*.

Citizens United (2010) *Plants of southern New Jersey: Plant profile for Solidago altissima (tall goldenrod)*. [www.cumauriceriver.org/.](http://www.cumauriceriver.org/)

Cummins, D. (2014) *Field-grown cut flowers for Luisiana*. Available at: [www.lsuagcenter.com/.](http://www.lsuagcenter.com/)

Hilty, J. (2015) *Wildflowers of Illinois in savannas and thickets: Coral-berry (Symphoricarpos orbiculatus), honeysuckle family (Caprifoliaceae)*. Available at: [http://www.illinoiswildflowers.info/.](http://www.illinoiswildflowers.info/)

Hurteau, M. (2006) *Plant guide for common yarrow (Achillea millefolium)*. USDA- Natural Resources Conservation Service, formerly USDA NRCS National Plant Data Center, c/o, Environmental Horticulture Department, University of California, Davis, California. Available at <http://plants.usda.gov/plantguide/pdf/cs_acmi2.pdf>

St. John, L., Tilley, D., Hunt, P., and Wright, S. (2012) *Plant guide for red fescue (Festuca rubra)*. USDA- Natural Resources Conservation Service, formerly USDA NRCS National Plant Data Center, c/o, Environmental Horticulture Department, University of California, Davis, California. Available at <https://plants.usda.gov/plantguide/pdf/pg_feru2.pdf>

Sullivan, J. (1992) *Dactylis glomerata. In: Fire effects information system*, [Online]. U.S. Department of Agriculture, Forest Service, Rocky Mountain Research Station, Fire Sci- ences Laboratory (Producer). Available: [http://www.fs.fed.us/database/feis/.](http://www.fs.fed.us/database/feis/)

Tenaglia, D. (2007) *Solidago altissima L.* Available at: [http://missouriplants.com/.](http://missouriplants.com/)
